# An unexpected tumour of the finger: review and management

**DOI:** 10.1080/23320885.2019.1573639

**Published:** 2019-02-14

**Authors:** N. Jumper, E. Caffrey, N. McInerney

**Affiliations:** a Department of Plastic, Reconstructive and Hand Surgery, Galway University Hospital, Galway, Ireland;; b Department of Histopathology, Galway University Hospital, Galway, Ireland

**Keywords:** Digital papillary adenocarcinoma, hand tumour, eccrine tumour, finger mass

## Abstract

We report the case of digital papillary adenocarcinoma in a patient presenting with a solitary fingertip mass. This rare sweat gland tumour has a frequently inconspicuous clinical course but significant potential for recurrence and metastasis. The prognostic implications therefore highlights the necessity of addressing even benign-appearing lesions with expedience.

## Introduction

Digital papillary adenocarcinoma (DPA), a cutaneous tumour thought to be of eccrine origin, was first described by Helwig in 1979 and published in 1984 as “eccrine acrospiroma” [1]. His contribution to the first case series, published by Kao et al in 1987, described two entities: aggressive digital papillary adenoma (ADPA) and adenocarcinoma (ADPAca), providing the first detailed clinicohistopathological reference for this neoplasm [2]. An updated retrospective analysis of this series, published with additional data in 2000 by Duke et al, determined none of the clinical or histologic parameters were predictive of biologic behaviour and therefore all tumours were to be considered potentially malignant, leading to one designation where the term ‘aggressive’ is omitted: digital papillary adenocarcinoma [3]. The often cystic and clinically indolent nature of DPA in addition to 85% of tumours occurring on the hands, of which 79% present on the fingers, frequently leads to misdiagnosis as a benign lesion [3]. The experience communicated in several case reports, as well as smaller case series, support the findings of these publications, demonstrating high rates of local recurrence (up to 50%) and metastasis (14–41%) [3]. Despite these accounts in the literature, the paucity of cases of this rare tumour leave definitive guidelines for management lacking. Here we report the case of presumed ganglion cyst or glomus tumour subsequently revealed to be a digital papillary adenocarcinoma on histopathology, thereby necessitating a review of the literature to determine the most appropriate intervention.

**Table 1.** Table detailing the antibodies used for immunohistochemistry of our patient tissue sections and the associated target layer within the tissue.

Antibody	Expression	Location of staining
Actin	Positive	Myoepithelial
Calponin	Positive	Myoepithelial
p63	Positive	Myoepithelial
D2-40 (podoplanin)	Positive	Myoepithelial
CK5/6	Positive	Myoepithelial & Epithelial
AE1/3	Positive	Epithelial
EMA (epithelial membrane antigen)	Positive	Epithelial
BerEp4	Weakly positive	Epithelial
S100	Patchy expression	Epithelial
CEA (carcinoembryonic antigen)	Negative	n/a

## Case report

A 67-year old right-hand-dominant male was referred to our plastic surgery department with a 3 year history of a swelling on his right middle fingertip with no prior history of trauma to the digit. The mass had been slowly enlarging over the preceding 3–4 months, was becoming increasingly tender and occasionally bled due to a superficial abrasion on the surface. The patient had a past medical history of Grave’s disease, atrial fibrillation and a perforated gastric ulcer.

**Figure 1.**
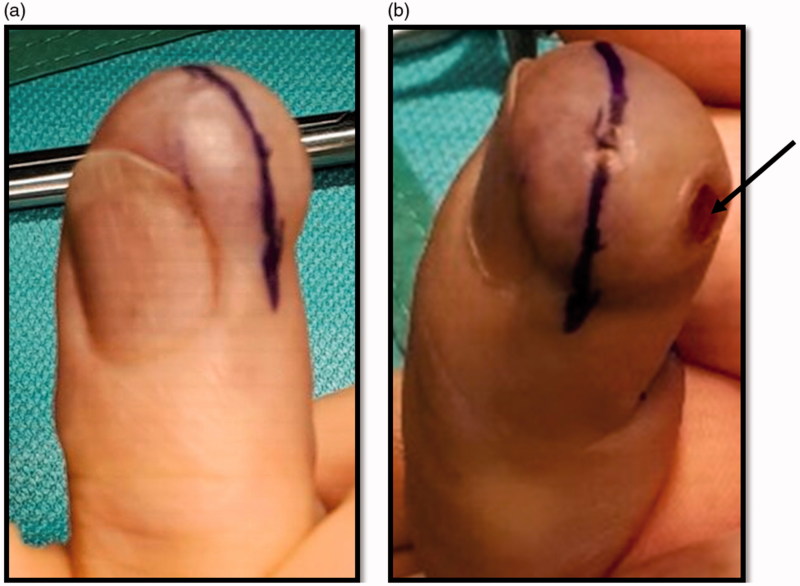
(a,b) Pre-operative images of the distal right middle finger volar surface. Figure b shows the surface ulceration evident at presentation (black arrow).

Examination revealed a 2 × 1.5cm mass over the distal pulp and hyponychium of the right middle finger. The swelling was mildly tender and there was an area of superficial ulceration centrally (Figure 1). The digit was neurovascularly intact with normal range of motion and no evidence of local erythema, inflammation or discolouration. The working differential diagnosis following initial consultation included glomus tumour, ganglion cyst and haemangioma, thus the patient was booked for surgery without prior imaging.

**Figure 2.**
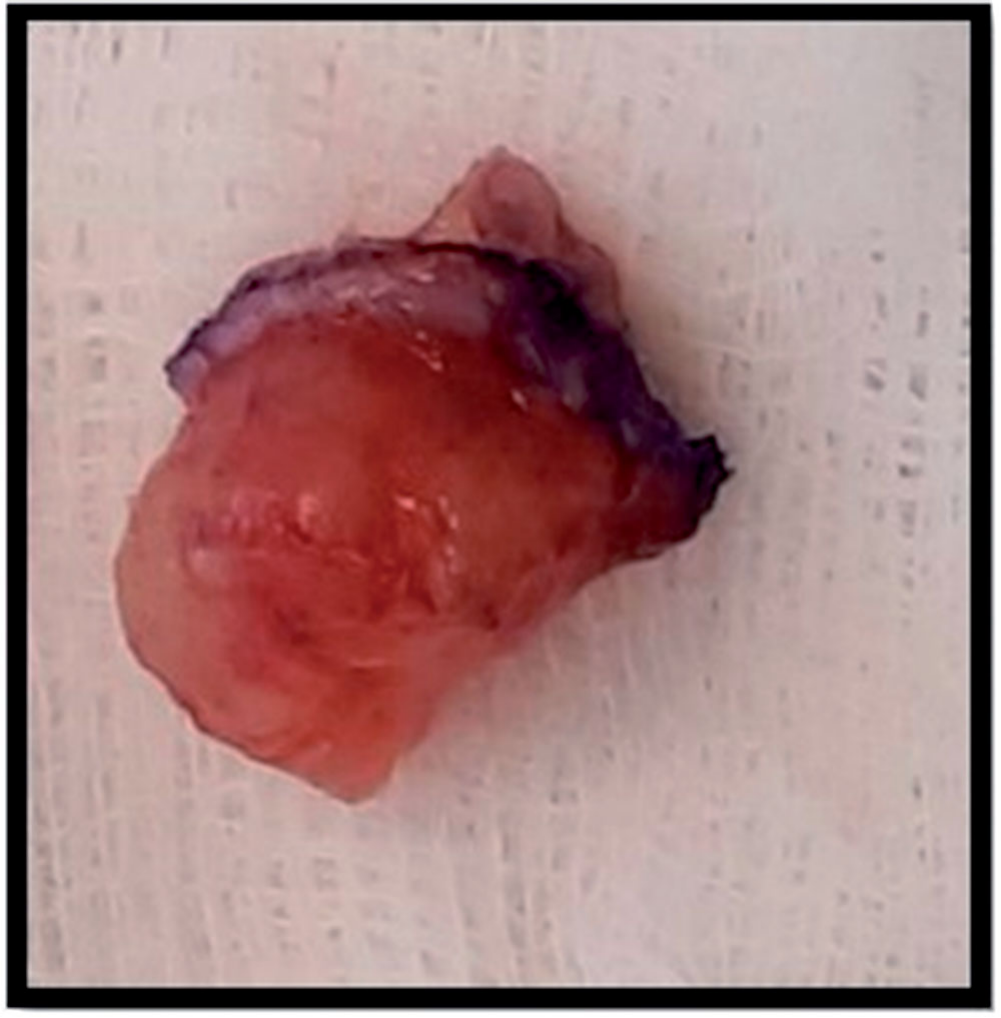
Intraoperative image of specimen excised from right distal fingertip.

**Figure 3.**
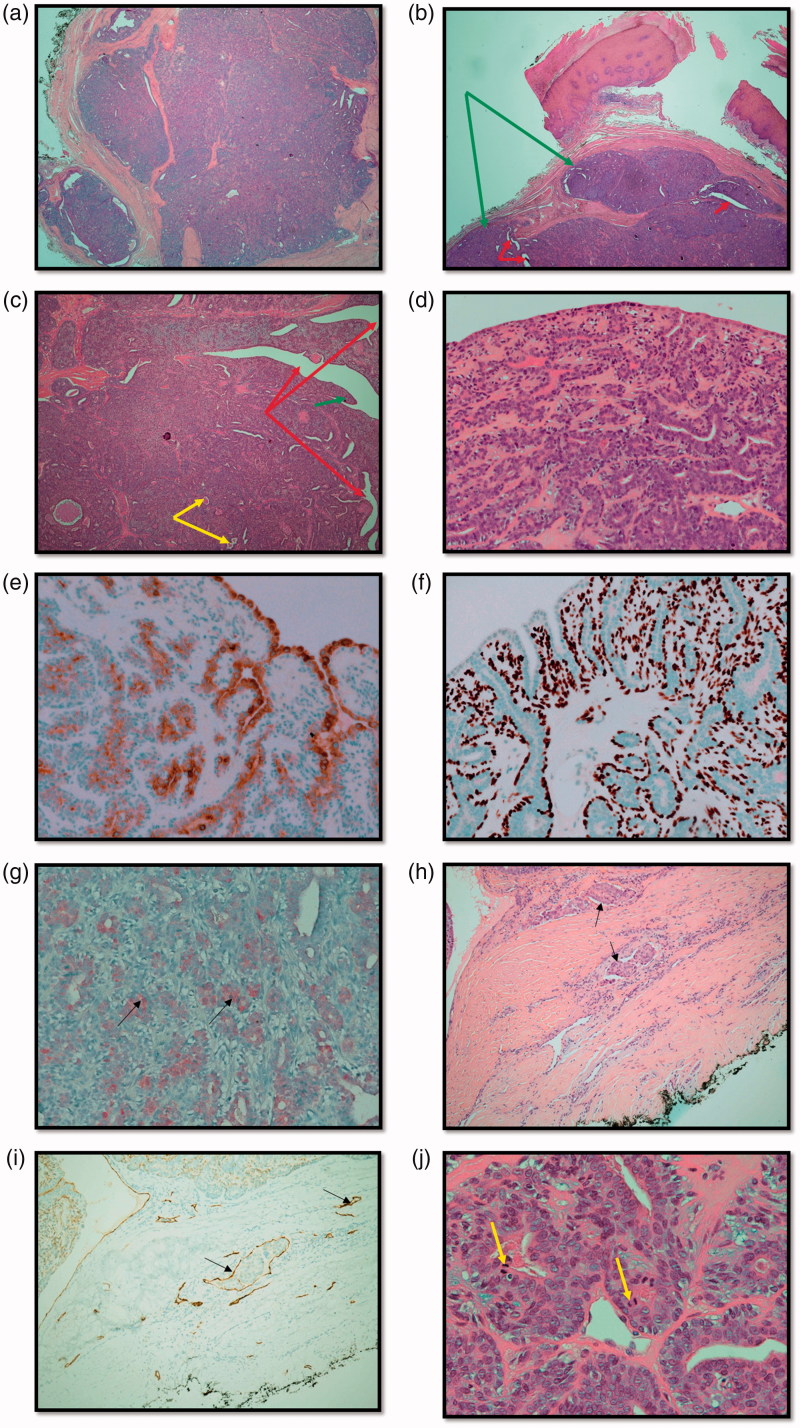
(a) Haemotoxylin and Eosin (H&E) stained section showing an overview of the multinodular, circumscribed and predominantly solid tumour (x2). (b) H&E stained section demonstrating multinodular tumour (green arrows) that is largely solid but with and ductal/tubular structures (red arrows) (x2). (c) H&E section demonstrating cystic spaces (red arrows) with papillary projections (green arrows) and tubules (yellow arrows) (x4). (d) H&E section showing double epithelial layer (x20). (e) Epithelial membrane Antigen (EMA) stain positivity. Expressed by ductal epithelial cells (x20). (f) p63 myoepithelial marker stains positive (x20). (g) S-100 stains weakly positive (black arrows) (x20). (h) H&E stained section demonstrating lymphovascular invasion (LVI) (black arrows) (x10). (i) D2-40 (podoplanin) stained section demonstrates lymphatic endothelium (black arrows) supporting the evidence for LVI (x10). (j) Mitotic figures indicated by yellow arrows (x40).

One month following referral, the patient underwent excision biopsy of the lesion which was not found to be communicating with bone, nerve or tendon and the intra-operative impression was that of a ganglion cyst (Figure 2). The initial histopathology examination reported a circumscribed multinodular predominantly solid tumour (Figure 3(a,b)) ulcerating the overlying epidermis. There were focal papillary projections with tubular/ductal structures (Figure 3(c)) where ducts were lined by a double layer of epithelium consisting of inner cuboidal cells and an outer myoepithelial layer (Figure 3(d,e,f,g)). There was also evidence of lymphovascular invasion (Figure 3(h,i)), focally high mitotic activity (Figure 3(j)) and the tumour was shown to abut the margins. Immunohistochemistry was performed (Table 1) confirming a myoepithelial cell population expressing smooth muscle actin, calponin, D2-40 (Figure 3(i)) and p63 (Figure 3(f)). The tumour was classified as digital papillary adenocarcinoma. The histopathology sections were sent to an external institution for expert opinion and the diagnosis affirmed with recommendation of complete excision and close follow-up. This recommendation was echoed when the case was discussed at the local multi-disciplinary meeting.

**Figure 4.**
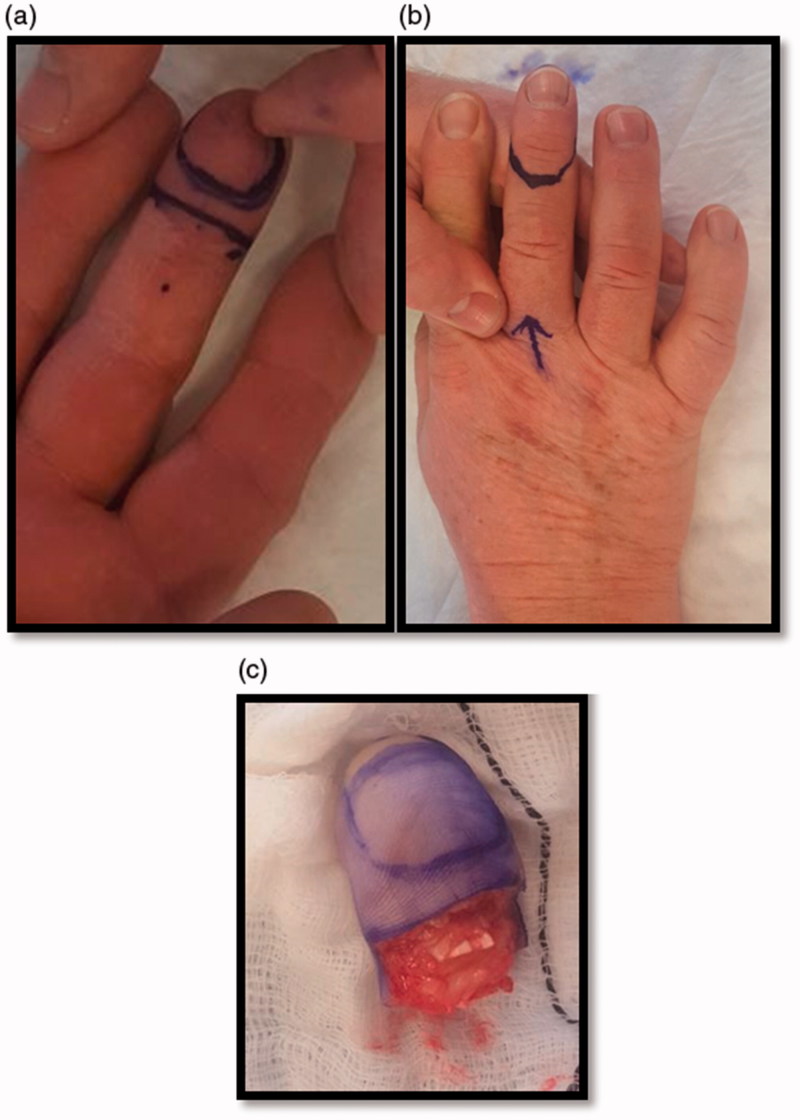
(a,b) Photographs indicating the area for excision and appropriate margin for conservative partial amputation through the DIPJ. (c) Photograph of the amputated piece sent for histological confirmation of complete excision.

The patient underwent partial amputation of the digit (Figure 4) and computed tomography (CT) scan of thorax, abdomen and pelvis, which showed no evidence of distant disease. The histology report on the wider excision specimen confirmed no invasion of underlying bone and margins free of tumour (residual tumour in wider specimen 0.5 mm). This patient is currently 1 year disease-free and will be followed closely in the outpatient department with clinical examination, yearly chest X-ray and additional imaging as needed for at least a ten year period.

## Discussion

Digital papillary adenocarcinoma (DPA) is a rare sweat gland tumour found predominantly on the volar aspect of the fingers. There is a higher prevalence in men, occurring primarily between the fifth and seventh decades [4,5]. The digital location, in addition to a typically indolent course, commonly results in the misdiagnosis of DPA as a benign lesion leading to delayed intervention and potentially worse prognosis. The failure of reliance on both histopathological parameters and clinical behaviour to predict either recurrence or metastasis has led to consensus within the literature to regard all DPA as malignant, abandoning previous divisions of this entity into benign adenoma and its pernicious counterpart, adenocarcinoma [3].

The frequently inconspicuous clinical presentation has led to a plethora of differential diagnoses at presentation, ranging from benign lesions (ganglion cyst, giant cell tumour, glomus tumour, foreign body granuloma, soft tissue infection or lipoma) to more sinister interpretations (SCC, melanoma or metastatic deposit) [2]. Histologically, DPA is fundamentally characterised by the presence of papillary projections into cystic spaces in a mixed solid-cystic multinodular tumour, lined by a double layer of epithelial cells [2,3,6,7]. Cytologic atypia is usually not marked. Immunohistochemistry is of limited value to differentiate DPA from histologically similar benign adnexal tumours but both p63 and D2-40 can be useful in its discrimination from metastatic adenocarcinoma to the skin [7,8]. Non-malignant differentials such as hidradenoma and apocrine cystadenoma are rare in digits and the histologically similar papillary eccrine adenoma histology shows symmetry with no cellular atypia [6]. Where distant-to-cutaneous metastatic papillary adenocarcinoma cannot be ruled out, screening for a primary lesion in the thyroid (ultrasound), breast (thorough examination) and gastrointestinal system (OGD, colonoscopy) is prudent.

As in our case, complete work-up for DPA is frequently undertaken after initial excision when the histological diagnosis confirms an unexpected malignancy necessitating further investigation and a considered treatment approach. Based on the available literature we considered our management strategy for primary DPA to be three-pronged. First, complete excision of the tumour. With no available guidelines on surgical excision margins we based our decision to perform conservative partial amputation of the distal right middle finger on extrapolated data from similar neoplasms, a review of treatment recommendations in the literature and histopathology results of the first specimen, which showed positive resection margins, lymphovascular invasion but no bony involvement. Duke et al. reported that aggressive re-excision can reduce local recurrence rates to 5%, whereas up to 50% recurred when left incompletely excised [3]. Excision methods vary significantly between reported cases, including Mohs Micrographic Surgery (MMS), wide local excision (WLE), partial amputation and even complete ray excision [9]. Despite the survey by Hsu et al suggesting no benefit of amputation over WLE, we felt the histological identification of lymphovascular invasion and high mitotic rate in our case warranted more aggressive intervention [4,10]. Additionally, we were concerned that any form of graft or flap reconstruction following WLE could potentially delay identification of future signs of early recurrence.

The second aspect of our management approach was staging of the tumour. We were satisfied that our patient, with a negative CT of the thorax, abdomen and pelvis, had no metastases. However, there are several reports advocating CT PET, MRI brain, bone scintigraphy and sentinel lymph node biopsy (SLNB) [11]. The role for SLNB in the literature is controversial [5]. All reported cases of positive SLNB resulted in subsequently negative completion lymphadenectomy and there have been several cases (2–17%) of distant metastasis in the absence of regional lymph node involvement [3,4,7]. Without evidence for improvement in long term survival the morbidity of a SLNB currently outweighs its utility in staging but this may change with further case series and research [12].

The final step in our management is vigilant long term follow-up. Although currently contentious, SLNB was initially performed for DPA in an attempt to identify patients with subclinical regional disease before the development distant metastasis, which carries a poor prognosis despite trials of chemotherapy. The rate of metastasis reported in the three largest series on DPA in the literature is 14%–26%, with the lung being the primary site of disease progression (71%) [2,3,7]. DPA has been shown to have a protracted disease course with some instances of recurrence delayed up to 20 years after the original presentation [3]. There is no standardised regimen for the use of systemic chemotherapy in cases of metastatic DPA, although several agents have been ventured. There was some initial success reported with the combination of carboplatin and paclitaxel but treatment was discontinued due to adverse effects [13]. We found only one case report in the literature regarding radiation and while there is no series or agreed framework reported, there may be scope for its employment in either neo-adjuvant or post-operative treatment [14]. There is general consensus within published cases that any metastatic disease amenable to surgery should undergo dissection or excision but given the poor response of disseminated disease to alternative treatment regimens, management should be largely pre-emptive with regular review and early detection of recurrence [4,12]. The minimum recommended follow-up period is ten years with thorough clinical assessment, yearly chest x-ray and judicious serial axillary ultrasound scans.

In summary, our patient, similar to many other reported cases, was referred on a non-urgent basis with a presumed cyst of the finger, which on histological examination following excision proved to be malignant. Digital papillary adenocarcinoma is a rare tumour with significant potential for recurrence and metastasis. To date, the majority of data for DPA originates in three large case series: Kao et al. [2] (*n* = 57), Duke et al. [3] (*n* = 67) and Suchak et al. [7] (*n* = 31), with remaining insight arising from scattered case reports in the literature. The rarity of this entity has meant that management is largely based on experiences within the literature and not standardised guidelines. It was based on the available literature that we determined our patient’s care: conservative partial amputation, staging and close follow-up. The case described here highlights the importance of early intervention for even benign-appearing lesions, which are the cases often left the longest on surgical waiting lists. Plastic surgeons should be mindful of these entities when allocating outpatient clinic appointments based on external referrals. The three-step management approach outlined in this article may prove useful to those searching the literature for guidance in the absence of standardised protocols.
